# Commentary: Beta-Band Oscillations Represent Auditory Beat and Its Metrical Hierarchy in Perception and Imagery

**DOI:** 10.3389/fnins.2016.00389

**Published:** 2016-08-23

**Authors:** Sundeep Teki, Tadeusz W. Kononowicz

**Affiliations:** ^1^Department of Physiology, Anatomy and Genetics, University of OxfordOxford, UK; ^2^CEA.DSV.I2BM.NeuroSpin - Institut National de la Santé et de La Recherche Médicale Cognitive Neuroimaging UnitGif sur Yvette, France

**Keywords:** timing and time perception, rhythm perception, music perception, magnetoencephalography, predictive coding, beta oscillations, beat perception

The ability to predict the timing of natural sounds is essential for accurate comprehension of speech and music (Allman et al., 2014). Rhythmic activity in the beta range (12–30 Hz) is crucial for encoding the temporal structure of regular sound sequences (Fujioka et al., 2009, 2012; Bartolo et al., 2014; Teki, 2014; Bartolo and Merchant, 2015). Specifically, the power of induced beta oscillations in the auditory cortex is dynamically modulated according to the temporal pattern of beats (Fujioka et al., 2012), such that beat-related induced beta power decreases after the beat and then increases preceding the next beat as depicted in Figure 1A. However, it is not known whether beta oscillations encode the beat positions in metrical sequences with physically or subjectively accented beats (i.e., “upbeat” and “downbeat”) and whether this is accomplished in a predictive manner or not.

**Figure 1:**
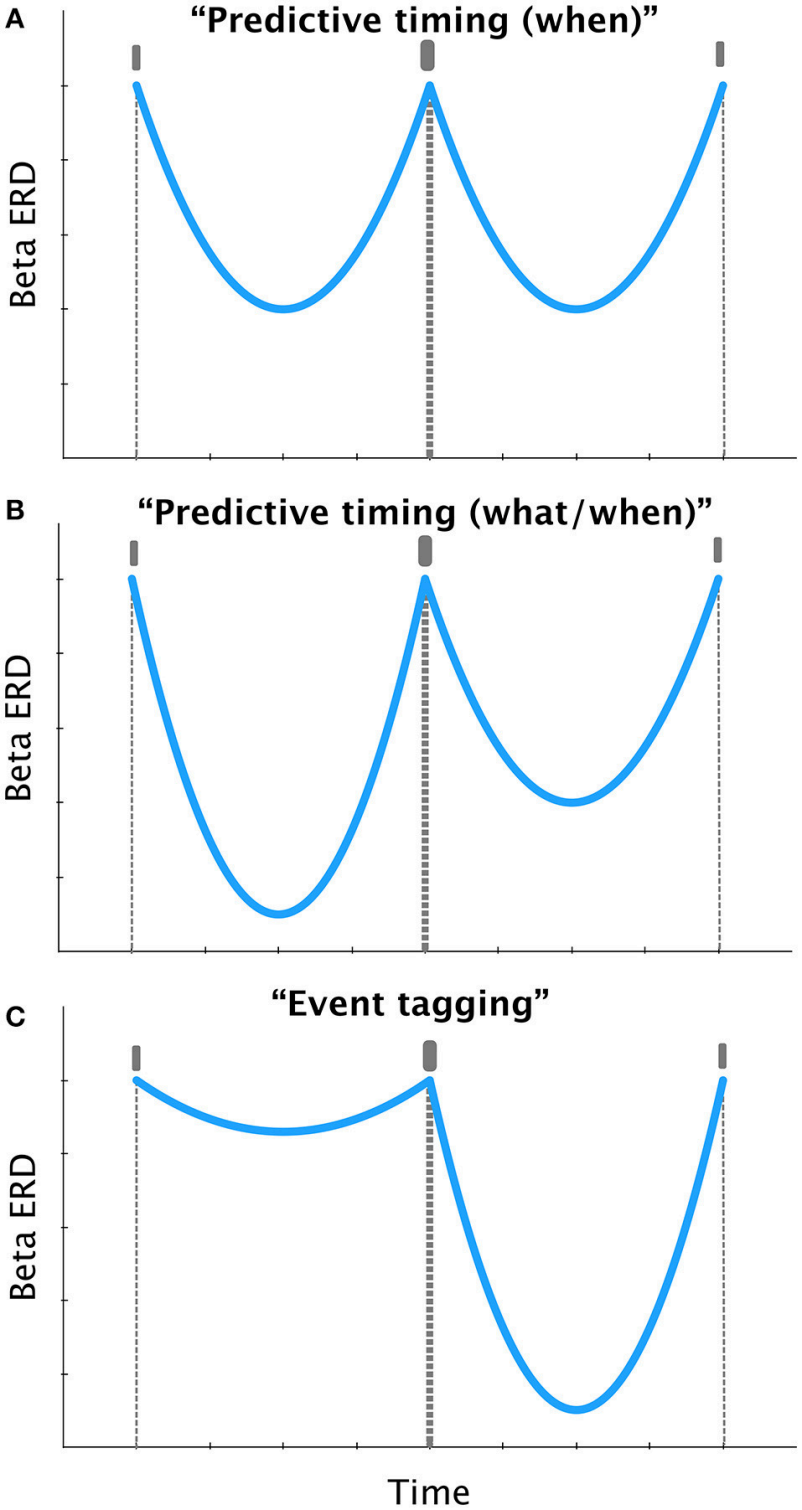
**Schematic depiction of the time course of induced beta oscillatory activity for a hypothetical sound sequence (indicated by vertical bars in gray, in the order, upbeat, downbeat, and upbeat), in accordance to the “predictive timing” and “event tagging” mechanisms**. Presented pattern is based on previous studies such as Fujioka et al. (2012). **(A)** Predictive timing theory (e.g., Arnal and Giraud, 2012) suggests that beta power should peak before each sound, such that the rebound of beta power could be predictive of the timing of the upcoming sound, regardless of the salience of the sound. **(B)** A hypothesized predictive code that also encodes the identity of the salient events in a sequence may show modulation of the stereotypical beta ERD response in panel **(A)**, expressed in terms of differential magnitude (here, greater beta suppression) before the salient event. As opposed to panel **(A)** beta power is not modulated in the same manner before upbeats and downbeats, allowing the encoding of “what” and “when” information in a manner consistent with the predictive timing framework (e.g., Arnal and Giraud, 2012). **(C)** Event tagging proposal (Iversen et al., 2009; Hanslmayr and Staudigl, 2014) suggests that beta power encodes accented events and should peak after the accented sounds, which is in contradiction with the predictive coding of “what” information depicted in panel **(B)**.

In a recent study, Fujioka et al. (2015) used magnetoencephalography to examine the role of induced beta oscillations in representing “what” and “when” information in musical sequences with different metrical contexts, i.e., a march and a waltz. Musically trained participants listened to 12-beat sequences of metrically accented beats, where every second (march) or third (waltz) beat was louder, along with unaccented beats at the same intensity. The paradigm consisted of two phases: A *perception* phase where accented beats were presented in march or waltz contexts and participants were required to actively perceive the meter, followed by an *imagery* phase where unaccented beats were presented at a softer intensity and participants had to subjectively imagine the meter.

Similar to their previous study (Fujioka et al., 2012), the authors found that event-related beta desynchronization (ERD) follows the beat, i.e., beta ERD response showed a sharp decrease after the stimulus, attained a minima with a latency of ~200 ms, and subsequently recovered with a shallow slope (see Figure 2 in Fujioka et al., 2015). This result has been previously demonstrated (Fujioka et al., 2009, 2012), and extended by other groups using electrophysiological recordings in humans (e.g., Iversen et al., 2009), and macaques (Bartolo et al., 2014; Bartolo and Merchant, 2015), as well as demonstrating a role for induced beta oscillations in time production (Arnal et al., 2015; Kononowicz and van Rijn, 2015). This result was valid for both the march and waltz conditions in the perception and even more importantly in the imagery phase, implying a top-down mechanism. Significantly, the authors claimed that the beta ERD response in the auditory cortex differentiates between the positions of the downbeat and the following beat (see Figures 3, 4 in Fujioka et al., 2015).

The novel result reported by Fujioka et al. (2015) is that the beta ERD response in auditory cortex can distinguish between accented beat positions in metrical sequences. However, the underlying mechanisms are far from clear. Fujioka et al. (2015) explain their results using predictive coding theory (Bastos et al., 2012) but an alternative “event tagging” mechanism (Iversen et al., 2009; see Repp, 2005 and Repp and Su, 2013 for a review on mechanisms for metrical processing), may also account for metrical interpretation of beat-based sequences.

We consider the results of Fujioka et al. (2015) in the light of these two mechanisms, i.e., predictive timing and event tagging (Figure 1). The predictive coding framework posits that beta oscillations are associated with anticipatory behavior and predictive coding (Arnal and Giraud, 2012). An internal model is established that conveys top-down predictions based on modulation of beta power with the goal of predicting the next event as shown in Figure 1A. However, if the predictive code were to also represent the identity of the salient event (i.e., what) in addition to its timing (i.e., when), one may hypothesize a modulation of the beta ERD response before the downbeat, which might be expressed in terms of differential magnitude as shown in Figure 1B. Such a response, that is specific to the downbeat would predict both the identity and timing of accented beats.

According to the event tagging framework (Hanslmayr and Staudigl, 2014), beta oscillations encode salient events (e.g., downbeat) as depicted in Figure 1C. Specifically, desynchronization of induced beta power may reflect memory formation (Hanslmayr and Staudigl, 2014) or an active change in sensorimotor processing (Pfurtscheller and Lopes da Silva, 1999). During rhythm perception, where encoding the beat in memory is critical (Teki and Griffiths, 2014, 2016) the structure of the metrical accents and salient events may be represented by the depth of beta desynchronization. Therefore, the largest beta desynchronization may be expected to occur after the downbeat (Figure 1C). In the present study, the amount of beta desynchronization was found to be largest after the accented tones. Therefore, the reported results are consistent with the event tagging framework, suggesting that subjectively and physically accented events invoke changes in the encoding of these events (Pfurtscheller and Lopes da Silva, 1999; Repp, 2005).

However, it is plausible that the predictive timing and event tagging mechanisms may operate in concert. To confirm this hypothesis, one needs to assess whether any prediction, implemented as beta rebound, occurs *before* the accented tones (Figure 1B). In the current study, it is difficult to determine whether there is robust beta synchronization before the accented tones. Careful observation of the results (Figures 3, 4 in Fujioka et al., 2015) suggests that beta power is not modulated before the downbeat in either of the two metrical conditions, neither in the perception nor in the imagery phase, except for a weak effect for the waltz-perception condition in right auditory cortex.

It is therefore not evident whether beta ERD carries predictive information about salient events, in addition to their timing. Therefore, future studies should also focus on other (non-sensory) brain regions, like the supplementary motor area or basal ganglia that are implicated in encoding rhythmic patterns (Grahn and Brett, 2007; Teki et al., 2011; Crowe et al., 2014; Merchant et al., 2015), as it is possible that different regions may recruit distinct mechanisms.

Overall, this study has provided significant insights about the neural representation of musical sequences. Beta oscillations have repeatedly been shown to track the timing of events in sound sequences but whether they can differentiate between beat positions, i.e., also encode categorical information about the events has been highlighted by the present study. It is important to build upon the current results and identify the precise role of beta oscillations with respect to encoding of “when” and “what” information in natural sound sequences, and future research may benefit highly from the current study.

## Author contributions

All authors listed, have made substantial, direct and intellectual contribution to the work, and approved it for publication.

### Conflict of interest statement

The authors declare that the research was conducted in the absence of any commercial or financial relationships that could be construed as a potential conflict of interest.
